# A new perspective on behavioral inconsistency and neural noise in aging: compensatory speeding of neural communication

**DOI:** 10.3389/fnagi.2012.00027

**Published:** 2012-09-25

**Authors:** S. Lee Hong, George V. Rebec

**Affiliations:** ^1^Department of Biomedical Sciences, Ohio UniversityAthens, OH, USA; ^2^Department of Psychological and Brain Sciences, Indiana UniversityBloomington, IN, USA

**Keywords:** noise, energetics, aging, compensation, neural oscillations

## Abstract

This paper seeks to present a new perspective on the aging brain. Here, we make connections between two key phenomena of brain aging: (1) increased neural noise or random background activity; and (2) slowing of brain activity. Our perspective proposes the possibility that the slowing of neural processing due to decreasing nerve conduction velocities leads to a compensatory speeding of neuron firing rates. These increased firing rates lead to a broader distribution of power in the frequency spectrum of neural oscillations, which we propose, can just as easily be interpreted as neural noise. Compensatory speeding of neural activity, as we present, is constrained by the: (A) availability of metabolic energy sources; and (B) competition for frequency bandwidth needed for neural communication. We propose that these constraints lead to the eventual inability to compensate for age-related declines in neural function that are manifested clinically as deficits in cognition, affect, and motor behavior.

## Introduction

Almost unequivocally, aging results in two primary declines in cognitive and motor behavior. First, there is a general slowing of both movement and cognitive reactions. Second, aging results in an increase in intra-individual variability, often termed behavioral inconsistency. A widely held view is that the source of behavioral inconsistency in aging is neural noise, that is, an increased presence of random background activity in brain signals (Li et al., 2001; Li and Sikstrom, 2002). What results is the flattening of neuronal activation profiles, leading to greater variability in neural responses to external signals (Li and Sikstrom, 2002). When extended to behavior, these variable patterns of neural activity will lead to more variable cognitive responses (e.g., Bunce et al., 2004; Nesselroade and Salthouse, 2004; Williams et al., 2005).

When brain activation patterns are viewed as discrete events, there is indeed a reduction in the speed of brain activity with aging. Using event-related potentials (ERP) such as P300, reports have consistently shown that aging leads to increased latency in brain activation that can be associated with slowing of behavioral responses. In response to external stimuli during cognitive tasks, for example, the elderly exhibit a decrease in the amplitude of the event-related potential and an increase in latency (Rossini et al., 2007 for a review). Indeed, these changes can be linked to the slowing of nerve conduction velocities with age that are a consistent finding in the literature (Peters, 2002).

Contrary to the ERP data, slowing is not a universal phenomenon of aging, if brain activity is viewed in terms of brain rhythms or neural oscillations (Duffy et al., 1993; Barnes, 1994). Instead, aging has been shown to alter the spectral composition of electroencephalographic (EEG) activity, resulting in changes to both resting and task-related EEG. For example, aging leads to a reduction in power within the theta band (4–7 Hz) during a word-recognition task (Cummins and Finnigan, 2007). In addition a stronger presence of resting theta rhythms has been shown to be associated with better memory, attention, and executive function in seniors (Finnigan et al., 2011). A synthesis of the aforementioned findings points to age-related declines during rest and while performing cognitive functions that is associated with the altered patterns oscillatory neural activity.

An important question that arises is whether there are potential unifying principles that can explain both increased neural noise and slowing of neural activity in conjunction with aging. The goal of this perspective paper is to propose the idea that compensatory speeding of neural activity through increased firing rates and oscillation frequency in the face of declining nerve conduction velocities accounts for the changes in EEG oscillations in aging. A secondary goal is to connect the metabolic cost and age-related declines in metabolic physiology with the energetic cost of increased neural oscillation frequency. Finally, we briefly provide links between this perspective and its potential clinical implications.

## Neural noise and compensatory activation

The central tenet of this perspective paper is that aging results in the compensatory speeding of brain activity in order to overcome delays in nerve conduction. Compensation in neural transmission at the cellular level has already been introduced in the past, as raised by the selective sparing of different aspects of the neuron in the face of neurodegeneration (Barnes, 1994). Here, we provide the potential for such compensatory changes due to aging to be reflected in EEG patterns and is revealed when the entire power spectrum is considered, rather than changes in specific brain rhythms.

Generally, frequencies above the high beta range (20–30 Hz), i.e., gamma, are often widely discussed in the study of brain rhythms in aging. Yet, what is often not included in the discussion of compensatory processes in the aging brain is the potential of compensatory speeding of brain activity due to delays in the conduction of neural signals. Consonant with increased neural noise, the re-distribution of spectral power to higher frequencies will result in a flatter power spectrum. Based on the existing literature, one can expect two age-related changes in the EEG power spectrum. First, there should be an increase in the proportion of delta activity in the low frequency, 1–3 Hz range (Klimesch, 1999; Rossini et al., 2007). Second, there should be a flattening of the power spectrum at 4 Hz and above, with an increased presence of high frequency activity (Klimesch, 1999; Rossini et al., 2007). When viewed from the perspective of the entire power spectrum, these two age-related changes in the power spectrum will be reflected in a rapid decay in power beyond the delta band. The overall signal in the time domain thus has a strong presence of slow frequency waves through the signal, while also being more unpredictable in time.

Indeed, not all broad spectrum activity, as indicated by a flat distribution of power, has to necessarily be a reflection of random noise. There are other viewpoints that consider the irregular patterns of variability in brain activity to be unrelated to random events. Instead, the irregular patterns of neural activity serve a functional purpose, in particular, for information transmission and efficient neural coding (Softky and Koch, 1993; Stevens and Zador, 1995, 1998; Shadlen and Newsome, 1998). From the perspective of functional irregularity, the variability in neural spikes is considered to be the reflection of highly precise temporal firing (Stevens and Zador, 1998). Rather than reflecting irreproducible random patterns, the variable patterns of neural activity can be reproduced through mathematical models (Softky and Koch, 1993).

Yet, regardless of whether one chooses to take the noise or functional perspective on irregularity in brain activity, in both cases, the power spectrum will appear flat, with power more evenly distributed into the higher frequencies. From a purely data-driven standpoint, there would be little to distinguish the two sources of variability. This allows us to take a unifying perspective on the flattening of the neural oscillation power spectrum in aging that connects both functional and random viewpoints of irregular brain activation patterns.

**Figure 1:**
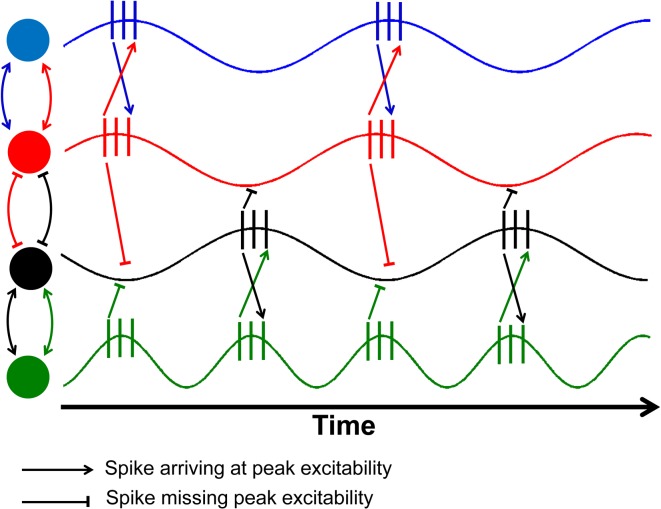
**Schematic illustration of the effects of phase shifts and compensatory speeding.** This figure is an adaptation from Fries (2005) as a demonstration of the role of conduction delays leading to a slight phase shift between electrical signals from two neurons, denoted by different colored circles. The relationship between the red and black nodes have been delayed so much that the spikes from one neuron arrive at the valley in excitability of the other. The green neuron has double the firing rate of the black neuron, allowing half of the spikes to arrive at peak excitability.

The key concept that connects both the random and functional views of brain activation variability has to be constructed on the basis of neural communication through coherence or synchrony between signals transmitted across the brain (Klimesch, 1999; Fries, 2005; Babiloni et al., 2006). Within this conceptual framework, different brain regions communicate through synchronized signals that are linearly correlated in order to transmit neural information. Normally, the two signals in question will often exhibit a slight phase difference due to naturally occurring nerve conduction delays that are often much smaller than a whole cycle (Fries, 2005). Knowing that age-related declines in nerve conduction velocity have been widely documented in the literature, leading to the slowing of neural communication (Peters, 2002), the transmission of an individual spike will begin to “drift” up to a point where spikes no longer arrive at the point of peak excitability (Figure 1).

Once the phase delays are sufficiently large, the spikes from the two signals will arrive at the low point or valleys in excitability (red and black neurons in Figure 1). Sensibly, one would hypothesize that one way to accommodate age-related nerve conduction delays would be to increase the firing rate of the neurons within the two regions. Once the phase difference between the two signals exceeds an entire cycle, then compensatory speeding will be needed to overcome the time lag in communication. As a result, one would expect a shift toward increased firing rates so that the spikes can once again coincide with peak excitability (black and green neurons in Figure 1).

Such increases in firing rates would be accompanied by spectral power that also becomes more broadly distributed, especially at higher frequencies. This is expected to occur as the change in nerve conduction velocity is unlikely to be uniform across all neurons, and thus, compensatory increases in firing frequency will appear as broad power spectrum rather than specific peaks on the power spectrum. Essentially, the aforementioned effect of compensatory speeding of neural activity will also lead to more irregular firing patterns, with data patterns that can just as easily be interpreted as increased neural noise.

## Compensation and energy consumption—synthesizing fMRI and EEG findings

More recent research surrounding compensatory activation in aging has utilized functional magnetic resonance imaging (fMRI) as the neuroimaging method of choice. A series of different experiments have found increased brain activation levels in the elderly during cognitive and motor tasks (e.g., Grady et al., 2005; Heuninckx et al., 2008; Zöllig and Eschen, 2009). Interestingly, the elderly that have better task performance recruited different brain regions than either young or old subjects who performed poorly on the task. What emerged from this research is that in order to maintain performance, some elderly subjects were able to recruit unrelated brain-regions to accomplish the task. A viewpoint counter to the compensation hypothesis is that of dedifferentiation (e.g., Carp et al., 2011; Dennis and Cabeza, 2011; Goh, 2011), which considers increased brain activation in the elderly as a marker of age-related decline. In this case, a loss of specificity in brain activation results in the spreading of neural activity to nearby regions, enlarging the areas that are activated during a given task. Regardless of whether one chooses the compensatory or dedifferentiation view of neural activation in aging, the common result is an increase in the amount of activity recorded by the MRI.

The primary connection between the EEG and fMRI approaches is that of energy expenditure. In short, what fMRI provides is an index of change in blood oxygen levels in specific brain regions over a period of time as well as blood flow. Essentially, fMRI measures brain oxygen consumption associated with glucose metabolism and the conversion of glutamate to glutamine (Sibson et al., 1998). In grey matter, increasing neuronal firing rate will result in a concomitant increase in energy consumption (Attwell and Laughlin, 2001). Despite the large differences in spatial and temporal resolution of both imaging approaches, increased activity in the fMRI reflects potential increases in oscillation frequency in EEG.

In aging, what is often observed in fMRI is an increase in number of brain areas that are activated to complete a given task in comparison to young subjects. These changes have been interpreted primarily from two perspectives: (1) dedifferentiation; and (2) compensation. The first viewpoint considers that as we age, the regions within the brain begin to lose specificity. As grey matter is lost overall, activity from the primary region involved in the task begins to spill over to neighboring regions. Thus, portions of the nearby regions that were normally reserved for other functions become active in order to compensate for declines in brain volume. From the viewpoint of compensation, increased activation of brain regions in the elderly reflects active recruitment of related brain regions to meet task demands. Instead of reflecting a spillover to neighboring regions, this increase in activity is a specific recruitment of other regions to support the completion of the task.

Regardless of which viewpoint one chooses (i.e., noise vs. functional variability; compensation vs. dedifferentiation), the common factor across all the viewpoints is that an overall increase in brain energy consumption with aging is to be expected. Knowing that a majority of the energy demands in the brain are subsumed by firing frequency (Attwell and Laughlin, 2001), there should, as a result, be an increase in metabolic demand in the aging brain.

## Energy limits to compensation: declines in mitochondrial function in aging

With increasing age, it is clear that some individuals have the capacity to remain highly functional over others, as presented in the idea of a “cognitive reserve” (Stern, 2009). While the brain is capable of compensating for age-related changes, clinical problems will not be observable, leaving the individual in a sub-clinical state. Eventually, the potential for compensatory increases in firing rate will be limited by metabolic resources needed to support higher neural oscillation frequencies. The upper limits of the brain's metabolic capacity will be reached at some point, and thus, conduction delays can no longer be overcome through compensatory speeding of neural activity, which would logically lead to clinical manifestations of disordered cognitive and/or motor function. At minimum, this would explain increased fatigability in aging (Allman and Rice, 2002). In addition, the ergonomics literature shows that fatigue leads to greater intra-individual variability (Guastello and McGee, 1987), providing another link to behavioral inconsistency. Thus, although much of the fMRI literature indicates a positive role for compensatory neural activation, the energy cost of the compensatory activity can pose a problem under more demanding conditions where fatigability and variability could pose a risk (e.g., walking around a cluttered room).

While increased compensatory activity comes at an energy cost, it will not necessarily affect all individuals in the same way. Another component of neural aging is an inherent decline in metabolic processes due to declines in function of mitochondria (Shigenaga et al., 1994; Bishop et al., 2010), the energy producers of the human body. Effectively, the lack of available energy will prevent compensatory increases from taking place, or at least shorten the period over which compensatory activity can be sustained. As a result, individuals who are able to maintain a higher level of metabolic function, perhaps by engaging in regular exercise or physical activity, will be able to tolerate the energy costs of compensatory neural activity.

## Negative consequences of compensatory speeding of neural activity

One of the limits placed on the brain is the maximal frequency at which neurons will be able to fire due to metabolic capacity and energetics. However, the upper limits of oscillation frequency are not the only limitations on compensatory speeding of neural activity. Another problem posed by aging and compensatory speeding is that neural communication occurs within frequency bands reserved for specific functions. Different neural oscillations within specific frequency bands serve different cognitive or motor operations (Rolls and Treves, 2011). For example, neural oscillations within the beta range are often linked to attention while theta rhythms are linked to memory recall (Stevens and Zador, 1998; Buzsáki, 2006).

One can thus conceive of the EEG frequency spectrum using the analogy of frequency allocation for radio signal propagation. Within this context, different frequency bands are reserved for transmission of specific signals (e.g., TV broadcasting, AM/FM radio, and WiFi). One example would be a situation in which the signal transmission frequencies of AM radio signals are increased to a point at which it spills over into that of TV broadcasting. While the images might not be affected, the audio that should be connected to the images becomes altered. Another such example would be a situation in which FM radio signals are transmitted within the frequency bands reserved for garage door openers. Garage doors would open and close outside the control of the homeowner when the 5 o'clock news is being transmitted.

**Figure 2:**
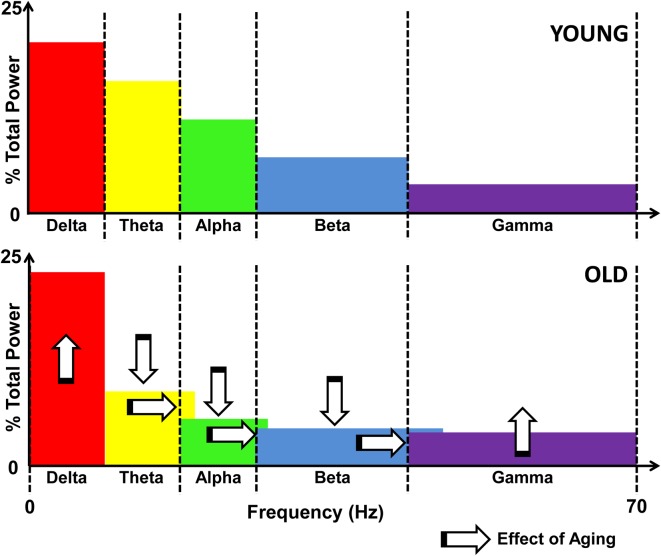
**Schematic illustration of the effects of aging on the EEG power spectrum.** Upper panel shows the distribution of power across the frequency spectrum, divided by bands in a young individual. The lower panel illustrates the effects of aging, namely, increased delta and gamma power, and decreased theta, alpha, and beta activity, as marked by vertical arrows. Also denoted is the spillover effect that results from compensatory speeding, denoted by the horizontal arrows as well as the overlapping color bands.

Applying the radio signal frequency allocation analogy to brain rhythms, the compensatory speeding of neural activation would lead to similar problems. The broadening of communication bands is an inherent part of the process of maintaining the balance between energy expenditure and entropy (unpredictability) of neural signals (Levy and Baxter, 1996; Tsubo et al., 2012). Specifically, the recent modeling efforts propose that the energy-entropy balance occurs by maximizing the entropy of neural firing rates under the constraints of energy consumption and uncertainty in the output being transmitted (Tsubo et al., 2012). Effectively, neurons seek to fire along as many frequency bands as possible while working within the two aforementioned constraints. Aging neurons not only need to continue to increase their oscillation frequencies to compensate for declining conduction, but, they also have to broaden the frequency bands along which communication occurs. Figure 2 illustrates the hypothesized effects of aging on the power spectrum of neural oscillations.

At the level of behavior and cognition, the broadening of these communication bands would result in unexpected shifts in emotion (e.g., negative thoughts) and cognition (e.g., memory lapses) as well as behavioral inconsistency in cognitive and motor performance. For example, if there is a spillover of beta rhythm activity into the gamma range, then neural activity related to the focusing of attention would tap into the oscillatory signals that normally reflect increased anxiety. Overlapping rhythms could be an explanation for the increased occurrence of depression and anxiety amongst seniors (Kastenschmidt and Kennedy, 2011). Furthermore, affective inconsistency in seniors has been linked to deficits across physical and cognitive domains (Strauss et al., 2002). Such patterns of altered emotion and cognition are consistent with the central thesis of our perspective that behavioral inconsistency in aging is a reflection of compensatory speeding of neural activity to overcome conduction delays that has been constrained by metabolic and neural communication bandwidth limitations.

## Summary and conclusions

In this paper, we outlined a new perspective on age-related changes in neural activity in a way that unifies contrasting viewpoints of neural noise and functional irregularity and variability. We present the possibility that compensatory increases in the frequency of neural oscillations to overcome decreased nerve conduction velocities will result in brain activity patterns that are more irregular and also resemble noise. By not taking either a functional or noise-related view of irregularity in brain activity, our “neutral” approach connects both the fMRI and EEG findings surrounding compensation and aging through the process of metabolic energy expenditure. Our perspective holds that limits on the brain's capacity to provide the necessary energetic sources to sustain the compensatory processes are a key component in the development of clinical symptoms in seniors. Finally, we raise the point that the spillover in neural oscillations from one band to another is what leads to unpredictable changes in motor behavior, cognition, and emotion in the elderly.

### Conflict of interest statement

The authors declare that the research was conducted in the absence of any commercial or financial relationships that could be construed as a potential conflict of interest.
